# Positive Youth Development Programs Targeting Students with Greater Psychosocial Needs: A Replication

**DOI:** 10.1100/tsw.2010.3

**Published:** 2010-02-12

**Authors:** Tak Yan Lee, Daniel T.L. Shek

**Affiliations:** ^1^ Department of Applied Social Studies, City University of Hong Kong, Shanghai, China; ^2^ Department of Applied Social Sciences, Hong Kong Polytechnic University, Shanghai, China; ^3^ Department of Sociology, East China Normal University, Shanghai, China; ^4^ Kiang Wu Nursing College of Macau, Macao

**Keywords:** subjective outcome evaluation, positive youth development, adventure-based counseling approach, volunteer training and services

## Abstract

The Tier 2 Program of the Project P.A.T.H.S. (Positive Adolescent Training through Holistic Social Programmes) targets adolescents with greater psychosocial needs, and the related programs were designed and implemented by school social workers. After completion of the Tier 2 Program (Secondary 1 Level), 9,931 participants in 212 schools responded to the Subjective Outcome Evaluation Form (Form C) in order to assess their views of the program, workers, and perceived effectiveness of the program. Based on the consolidated reports submitted by the agencies to the funding body, the research team aggregated the consolidated data to form a “reconstructed” overall profile on the perceptions of the program participants. Four major types of program were identified, including programs based on the adventure-based counseling approach (n = 58), programs concentrating on volunteer training and services (n = 31), programs offering both adventure-based counseling and volunteer training activities (n = 91), and other programs with different foci (n = 32). Results showed that high proportions of the respondents had positive perceptions of the programs and the workers, and over four-fifths of the respondents regarded the program as helpful to them. The present study provides support for the effectiveness of the Tier 2 Program of the Project P.A.T.H.S. in Hong Kong for the Full Implementation Phase.

## INTRODUCTION

The Project "P.A.T.H.S. to Adulthood: A Jockey Club Youth Enhancement Scheme" is a large-scale, positive youth development program designed for junior secondary school students (Secondary 1 to 3, i.e., Grades 7 to 9) in Hong Kong[1]. The word "P.A.T.H.S." denotes Positive Adolescent Training through Holistic Social Programmes. It consists of two tiers of program. The Tier 1 Program targets all students joining the program in a particular form (i.e., universal prevention initiative). Through the use of a structured curriculum, students learn competencies with reference to the 15 positive youth development constructs[2]. On the other hand, the Tier 2 Program is specially designed for students with greater psychosocial needs in different psychosocial domains (i.e., selective prevention). It is noteworthy that the researchers deliberately avoided using the term “at risk” because the term is very stigmatizing in the Chinese culture, and it deters parents and students from joining the related programs[3]. Based on the developmental needs of the students in a particular school, the school social work service providers design and implement the Tier 2 Program.

There are several characteristics of the Tier 2 Program in the project. First, the Tier 2 Program (Selective Program) targets adolescents with greater psychosocial needs who are identified in the Tier 1 Program and/or via other sources. It is recommended that information based on multiple sources, including objective assessment tools (e.g., Family Assessment Instrument, Life Satisfaction Scale, Hong Kong Student Information Form), teachers’ ratings, student records, and other relevant quantitative and qualitative information based on systematic assessment, will be used to identify students for the Tier 2 Program. Second, it is suggested that particular attention should be paid to students with special needs in the academic, personal (e.g., adjustment, mental health, and value concerns), interpersonal, and family domains. As a general guideline, it is expected that at least one-fifth of the adolescents and/or their parents of the Tier 1 participants will participate in the Tier 2 Program. However, under some special circumstances (e.g., many students have value confusions over material possession and difficulty in financial management), a Tier 2 Program targeting more than one-fifth of the students at each form could be designed within the available financial resources. Based on the above general guidelines, for those schools providing the full Tier 1 Program (i.e., 40 curriculum units in a 20-h program), they were expected to serve at least one-fifth of the students at each form. On the other hand, for schools providing the core Tier 1 Program (i.e., 20 curriculum units in a 10-h program), they would have to serve at least two-fifths of the students at each form.

For students identified as having special needs, they and/or their families will receive services based on the Tier 2 Program. To create more flexibility for the workers and in view of the diverse needs of the students, the NGO applicants have the choice of designing appropriate programs that target the needs of the students with reference to the positive youth development constructs, goals, and objectives covered in this project. Several nonmutually exclusive examples for the Tier 2 Program include (a) mentorship programs involving the alumni of the schools, (b) mental health promotion programs, (c) adventure-based counseling, (d) parenting programs, (e) service learning programs, and (f) resilience enhancement programs.

This paper presents and discusses the findings of an outcome evaluation of the Tier 2 Program implemented in the Full Implementation Phase (FIP) of the Project in 2007/08 based on the perspective of the participants. Two similar studies were conducted to evaluate the Tier 2 Program implemented in three cohorts: (1) the Experimental Implementation Phase (EIP) of the project in 2005/06 (3,072 Secondary 1 students in 52 schools); (2) EIP in 2006/07 (2,542 Secondary 2 students in 49 schools); and (3) FIP in 2006/07 (13,194 Secondary 1 students in 207 schools[4,5]. Both studies found that roughly four-fifths of the respondents of the three cohort studies regarded the program as helpful to them.

Although it is very common for social work agencies to design programs for adolescents with greater psychosocial needs (e.g., adventure-based counseling, voluntary training program), systematic evaluation and documentation of program evaluation have been rarely found in the local social work literature[6]. This paper will present a systematic study on social work intervention with adolescents. Besides giving some thoughts on the quality of the program delivered, it was also hoped that this paper would also contribute to the development of evidence-based social work practice in Hong Kong. Actually, in the literature on positive youth development programs, many programs have been designed to help adolescents with greater psychosocial needs and the related evaluation findings have been documented[7,8,9].

## METHODS

### Participants and Procedures

A total of 213 schools joined the Secondary 1 Program of the Project P.A.T.H.S. in the second year of the FIP (2007/08 school year). In these schools, there were 15,494 participants involved in the Tier 2 Program, of which 13,032 students were identified by teachers, parents, and/or via self-administered questionnaires as having greater psychosocial needs and thus were invited to join the Tier 2 Program. The other 2,462 participants were the parents and teachers of those identified students. The mean number of participants joining the Tier 2 Program per school was 72.74 (range: 13–360). The average number of sessions provided per school (normally 1.5–3 h per session) was 22.71 (range: 8–120).

The participants were invited to respond to the Subjective Outcome Evaluation Form (Form C), which was developed by the research team[7], usually immediately after completion of the Tier 2 Program. Nevertheless, one school refused to submit Form C because the school atmosphere was poor after an accident that happened during the Tier 2 Program implementation. As a result, only a total of 9,931 forms (mean = 46.84 forms per school, range: 7–198) were received from 212 schools (total number of participants was 15,414). The overall response rate was 64.43%. There are three plausible reasons for the low response rate: (1) some participants withdrew from the Tier 2 Program before completion, (2) some participants were absent in the last session and did not complete the evaluation form, or (3.) some schools did not invite the adult participants to respond to the evaluation form.

On the day of data collection, the purpose of the evaluation was explained and the principle of confidentiality was repeatedly emphasized to the participants. The participants were asked to indicate if they did not want to respond to the evaluation questionnaire (i.e., “passive” informed consent were obtained). All participants responded to all scales in the evaluation form in a self-administration format. Adequate time was provided for the participants to complete the questionnaire. To facilitate the program evaluation, the research team developed an evaluation manual with standardized instructions for collecting the subjective outcome evaluation data[10]. In addition, adequate training was provided to the social workers during the 20-h training workshops on how to collect and analyze the data collected using Form C.

### Instruments

The Subjective Outcome Evaluation Form (Form C) was designed by Shek and Siu[10] with an aim to measure the perceptions of Tier 2 Program. There are seven parts in this evaluation form:

1. Participants’ perceptions of the program, such as program design, quality of service, appropriateness of the program, and interaction among the participants (eight items).
2. Participants’ perceptions of the workers, such as the preparation of the workers, professional attitude and knowledge, and interaction with the participants (eight items).
3. Participants’ perceptions of the effectiveness of the program, such as promotion of different psychosocial competencies, resilience, and overall personal development (eight items).
4. Things that the participants appreciated the most (open-ended question).
5. Opinion about the workers (open-ended question).
6. Things that the participants learned from the program (open-ended question).
7. Areas that require improvement (open-ended question).

After collecting the data, the social work service providers were requested to input the data in an EXCEL file developed by the research team that would automatically compute the frequencies and percentages associated with the different ratings for an item. When the providers submitted the reports, they were also requested to submit the soft copy of the consolidated data sheets. The data from all service providers were then aggregated to “reconstruct” the overall profile by the research team. Since some amendments were made during the program implementation (e.g., program cancellation due to insufficient participants), the delivered program content was reported again in the program report.

As the Tier 2 Programs were designed according to the various needs and the contextual situations of different schools, it is hard to compare their results directly. Therefore, an analysis on the program contents was conducted to identify the major program elements of the Tier 2 Programs for all participating schools. Three research assistants (one with a Bachelor’s Degree in Social Sciences, one with a Bachelor’s Degree in Psychology, and one with a Master’s Degree and several years of front-line social work experiences) categorized the program contents delivered by the social workers as indicated in the program reports submitted to the research team. The criteria were strictly set to categorize a program according to the program proposal or revised proposal, together with the actual activities.

## RESULTS

Reliability analysis with the schools as the unit of analyses showed that Form C was internally consistent: eight items related to the program (alpha = 0.99, mean interitem correlation = 0.90), eight items related to the worker (alpha = 0.99, mean interitem correlation = 0.92), eight items related to the effectiveness (alpha = 0.99, mean interitem correlation = 0.91), and 24 items based on the entire Form C (alpha = 0.99, mean interitem correlation = 0.86).

Results of the content analyses showed that three major types of program elements were identified, namely, the adventure-based counseling approach, volunteer training and services, and others. A program will be classified as containing the adventure-based activities and counseling (ABC) elements if and only if the espoused theory was matched with the theory-in-action[11]. Examples related to volunteer training and services were program planning and program organizing activities, role play, and drama. The most popular volunteer services in this study were visiting and providing services for elderly, children, mentally challenged people, and disadvantaged groups. The programs without elements of ABC or volunteer training and services were classified as “others”. Examples of this category included groups promoting learning skills, learning motivation, emotional competence, self-presenting skills, self-efficacy, self-understanding, self-confidence and resilience, military adventure training, social skills training program, therapeutic group with narrative therapy approach, and cognitive behavior therapy. An analysis of the programs delivered by the social workers showed that two types of program approaches were frequently adopted in the Tier 2 Programs. They were volunteer training and services and the use of the ABC approach. A total of 84.91% of all Tier 2 Programs delivered by the 212 secondary schools contained at least one of these two elements.

The characteristics of the Tier 2 Programs of the 212 secondary schools are presented in Table 1. The Tier 2 Program can be categorized into four types by the main types of program elements used or program approaches adopted. They are (1) the ABC approach together with volunteer training and services (Type A, 42.92%), (2) ABC only (Type B, 27.36%), (3) volunteer training and services only (Type C, 14.62%), and (4) other approaches (Type D, 15.09%).

The mean overall effectiveness of all the Tier 2 Programs of the 212 secondary schools ranged from 4.23 to 4.74 on a 6-point scale towards the positive side. Since the Project P.A.T.H.S. made use of 15 constructs as identified by Catalano et al.[12], the average number of constructs indicated in the reports for these programs ranged from 5.33 to 9.25 and the mode was 8 (Table 1).

Based on the participants’ answers to the closed-ended questions (quantitative data), there are several observations. First, over 80% of the respondents perceived that they participated in the Tier 2 Program in a positive manner (Table 2). For example, 86.42% of the participants indicated that they had much interaction with other participants; 87.87% of the participants indicated that they were satisfied with the service. Second, a very high proportion of the participants had a positive evaluation of the social workers (Table 3). For example, 90.36% of the respondents indicated that the worker(s) were well prepared for the program, 89.32% of the respondents indicated that the worker(s) had good attitudes; 90.42% of the

**TABLE 1**
**Summary Table of Program Characteristics and Effectiveness**

| Main Program Approach | Clientele | Average No. of Participants | Average Program Attendance (%) | Average No. of Program Aims Indicated in the Reports | Average No. of Constructs Indicated in the Reports | Mean of Overall Effectiveness |
|---|---|---|---|---|---|---|
| ABC approach and volunteer training and services (Type A) (n = 91) | a (n = 70) | 51.94 | 82.71 | 2.10 | 6.73 | 4.58 |
| | b (n = 15) | 69.13 | 85.93 | 2.27 | 7.13 | 4.56 |
| | c (n = 4) | 139.00 | 86.38 | 1.75 | 9.25 | 4.27 |
| | d (n = 2) | 47.50 | 86.00 | 2.00 | 6.00 | 4.53 |
| ABC approach only (Type B) (n = 58) | a (n = 47) | 52.13 | 80.84 | 2.09 | 5.98 | 4.55 |
| | b (n = 6) | 63.33 | 81.23 | 2.50 | 7.00 | 4.62 |
| | c (n = 1) | 196.00 | 91.50 | 3.00 | 8.00 | 4.57 |
| | d (n = 4) | 105.75 | 89.25 | 1.75 | 7.25 | 4.25 |
| Volunteer training and services only (Type C) (n = 31) | a (n = 23) | 53.09 | 83.13 | 2.30 | 6.30 | 4.56 |
| | b (n = 8) | 76.13 | 77.18 | 2.25 | 9.00 | 4.47 |
| Other approaches (Type D) (n = 32) | a (n = 23) | 44.00 | 82.53 | 2.00 | 6.13 | 4.62 |
| | b (n = 6) | 100.67 | 83.62 | 3.17 | 6.17 | 4.23 |
| | d (n = 3) | 140.33 | 92.77 | 2.33 | 5.33 | 4.74 |

*Note:* a = only students involved; b = students and parents involved; c = students and teachers involved; d = students, parents, and teachers involved.

participants revealed that they were satisfied with the worker(s). Third, as shown in Table 4, over four-fifths of the respondents perceived that the program had induced positive changes in them. For example, the participants had learned how to help themselves through participating in the program (87.14%), the services had enhanced their growth (85.45%), and they had learned how to solve their problems through participating in the program (86.61%).

Furthermore, Table 5 shows that various domains of the subjective outcome evaluation were inter-related; the overall views about the program and the overall views about the workers were significantly correlated with each other, and both of them were positively correlated with all eight aspects of the perceived effectiveness of the program.

Comparisons between different program approaches were carried out by conducting one-way between-groups analyses. Two sets of analyses were carried out to address the questions of whether there were differences in the subjective outcome evaluation ratings between (1) programs with and without ABC elements and (2) programs with and without volunteer training and services. As shown in Table 6, analyses showed that the two types of programs did not differ in each category. A third set of analyses was

**TABLE 2**
**Comparison of the Positive Views towards Tier 2 Program among Different Program Approaches**

| | Participants with Positive Responses in Different Program Approaches | | | | | | | | | |
|---|---|---|---|---|---|---|---|---|---|---|
| | ABC Approach and Volunteer Training and Services[1] | | ABC Approach[2] | | Volunteer Training and Services[3] | | Others[4] | | Overall | |
| | n (Total Response) | % | n (Total Response) | % | n (Total Response) | % | n (Total Response) | % | n (Total Response) | % |
| The activities were carefully planned. | 3679 (4283) | 85.90 | 2451 (2856) | 85.82 | 1204 (1453) | 82.86 | 1119 (1283) | 87.22 | 8453 (9875) | 85.60 |
| The quality of the service was high. | 3658 (4272) | 85.63 | 2425 (2855) | 84.94 | 1179 (1452) | 81.20 | 1125 (1312) | 85.75 | 8387 (9891) | 84.79 |
| The service provided could meet the participants' needs. | 3713 (4278) | 86.79 | 2417 (2849) | 84.84 | 1213 (1451) | 83.60 | 1134 (1304) | 86.96 | 8477 (9882) | 85.78 |
| The service delivered could achieve the planned objectives. | 3698 (4268) | 86.64 | 2470 (2859) | 86.39 | 1214 (1451) | 83.67 | 1135 (1304) | 87.04 | 8517 (9882) | 86.19 |
| Participants could get the service they wanted. | 3598 (4269) | 84.28 | 2353 (2850) | 82.56 | 1194 (1451) | 82.29 | 1100 (1311) | 83.91 | 8245 (9881) | 83.44 |
| Participants had much interaction with other participants. | 3728 (4269) | 87.33 | 2441 (2844) | 85.83 | 1213 (1448) | 83.77 | 1137 (1297) | 87.66 | 8519 (9858) | 86.42 |
| Participants would recommend others who have similar needs to participate in the program. | 3484 (4261) | 81.76 | 2309 (2844) | 81.19 | 1145 (1449) | 79.02 | 1053 (1293) | 81.44 | 7991 (9847) | 81.15 |
| On the whole, participants were satisfied with the service. | 3779 (4273) | 88.44 | 2500 (2846) | 87.84 | 1224 (1445) | 84.71 | 1153 (1287) | 89.59 | 8656 (9851) | 87.87 |

[1] The program contents related to both the ABC approach and volunteer training and services were indicated in the Tier 2 Program reports.

[2] The program contents related to the ABC approach were indicated in the Tier 2 Program reports.

[3] The program contents related to volunteer training and services were indicated in the Tier 2 Program reports.

[4] Except the ABC approach and volunteer training and services, other program contents were indicated in the Tier 2 Program reports.

carried out to examine whether there were differences in the subjective outcome evaluation ratings between programs adopting two approaches, and programs adopting one approach or others. However, the results showed that participants attending programs that adopted both the ABC approach and volunteer training and services did not have significantly better views about the program than those attending programs adopting either one approach only or other approaches (Table 6).

**TABLE 3**
**Comparison of the Positive Views towards Tier 2 Program Workers among Different Program Approaches**

| | Participants with Positive Responses in Different Program Approaches | | | | | | | | | |
|---|---|---|---|---|---|---|---|---|---|---|
| | ABC Approach and Volunteer Training and Services | | ABC Approach | | Volunteer Training and Services | | Others | | Overall | |
| | n (Total Response) | % | n (Total Response) | % | n (Total Response) | % | n (Total Response) | % | n (Total Response) | % |
| The worker(s) had professional knowledge. | 3841 (4271) | 89.93 | 2534 (2857) | 88.69 | 1247 (1451) | 85.94 | 1175 (1288) | 91.23 | 8797 (9867) | 89.16 |
| The worker(s) demonstrated good working skills. | 3793 (4274) | 88.75 | 2515 (2852) | 88.18 | 1237 (1451) | 85.25 | 1147 (1288) | 89.05 | 8692 (9865) | 88.11 |
| The worker(s) were well prepared for the program. | 3886 (4262) | 91.18 | 2563 (2850) | 89.93 | 1268 (1449) | 87.51 | 1178 (1283) | 91.82 | 8895 (9844) | 90.36 |
| The worker(s) understood the needs of the participants. | 3763 (4265) | 88.23 | 2489 (2845) | 87.49 | 1239 (1448) | 85.57 | 1147 (1286) | 89.19 | 8638 (9844) | 87.75 |
| The worker(s) cared about the participants. | 3846 (4264) | 90.20 | 2517 (2852) | 88.25 | 1262 (1447) | 87.21 | 1164 (1284) | 90.65 | 8789 (9847) | 89.26 |
| The worker(s)' attitudes were very good. | 3863 (4268) | 90.51 | 2514 (2845) | 88.37 | 1251 (1446) | 86.51 | 1161 (1281) | 90.63 | 8789 (9840) | 89.32 |
| The worker(s) had much interaction with participants. | 3687 (4264) | 86.47 | 2407 (2852) | 84.40 | 1207 (1450) | 83.24 | 1118 (1284) | 87.07 | 8419 (9850) | 85.47 |
| On the whole, participants were satisfied with the worker(s). | 3900 (4266) | 91.42 | 2549 (2851) | 89.41 | 1272 (1449) | 87.78 | 1190 (1289) | 92.32 | 8911 (9855) | 90.42 |

## DISCUSSION

Several points can be highlighted in the study. First, Types B (ABC approach only) and C (volunteer training and services only) programs each took up about 15–27% of all programs, but the Type A program (a mixture of Types B and C) took up 42.9%. These two program approaches covered 84.9% of all delivered programs and were the dominant approaches adopted as compared with 86.6% in EIP 2005/06 for Secondary 1 students and 87.76% in EIP 2006/07 for Secondary 2 students[4,5]. They were both popular program approaches used by social workers working with adolescents and young people in Hong Kong. Both of them ground their theoretical base in the experiential learning theory[9,13,14]. The Type C program has a much longer history that dates back to the late 1960s and has since been a popular program, while Type B was formally adopted as the major program theory for a huge social intervention program entitled "The Understanding the Adolescent Project" (UAP) designed to combat the problems among students identified as adolescents-at-risk from 2001 to 2004 in Hong Kong[3,15].

**TABLE 4**
**Comparison of the Positive Views towards Tier 2 Program Effectiveness among Different Program Approaches**

| | Participants with Positive Responses in Different Program Approaches | | | | | | | | | |
|---|---|---|---|---|---|---|---|---|---|---|
| | ABC Approach and Volunteer Training and Services | | ABC Approach | | Volunteer Training and Services | | Others | | Overall | |
| | n (Total Response) | % | n (Total Response) | % | n (Total Response) | % | n (Total Response) | % | n (Total Response) | % |
| The service has helped participants a lot. | 3605 (4243) | 84.96 | 2355 (2820) | 83.51 | 1171 (1441) | 81.26 | 1069 (1276) | 83.78 | 8200 (9780) | 83.84 |
| The service has enhanced participants' growth. | 3692 (4240) | 87.08 | 2391 (2824) | 84.67 | 1184 (1438) | 82.34 | 1087 (1274) | 85.32 | 8354 (9776) | 85.45 |
| In the future, participants would receive similar service(s) if needed. | 3563 (4228) | 84.27 | 2303 (2809) | 81.99 | 1165 (1439) | 80.96 | 1050 (1273) | 82.48 | 8081 (9749) | 82.89 |
| Participants have learned how to help themselves through participating in the program. | 3741 (4236) | 88.31 | 2438 (2806) | 86.89 | 1212 (1438) | 84.28 | 1108 (1273) | 87.04 | 8499 (9753) | 87.14 |
| Participants have had positive change(s) after joining the program. | 3685 (4222) | 87.28 | 2393 (2806) | 85.28 | 1205 (1434) | 84.03 | 1095 (1270) | 86.22 | 8378 (9732) | 86.09 |
| Participants have learned how to solve their problems through participating in the program. | 3692 (4211) | 87.68 | 2422 (2803) | 86.41 | 1199 (1430) | 83.85 | 1090 (1258) | 86.65 | 8403 (9702) | 86.61 |
| Participants' behavior has become better after joining this program. | 3551 (4228) | 83.99 | 2268 (2813) | 80.63 | 1143 (1439) | 79.43 | 1024 (1271) | 80.57 | 7986 (9751) | 81.90 |
| Those who knew the participants agree that this program has induced positive changes in them. | 3469 (4231) | 81.99 | 2234 (2812) | 79.45 | 1138 (1439) | 79.08 | 1009 (1264) | 79.83 | 7850 (9746) | 80.55 |

**TABLE 5**
**Intercorrelations among Different Domains in Subjective Outcome Evaluation**

| Variables/Items | Views about Program | Views about Workers |
|---|---|---|
| Views about program | 1 | 0.93* |
| Views about workers | 0.93* | 1 |
| Perceived effectiveness of the program: | | |
| The service has helped participants a lot. | 0.91* | 0.88* |
| The service has enhanced participants' growth. | 0.89* | 0.86* |
| In the future, participants would receive similar service(s) if needed. | 0.91* | 0.83* |
| Participants have learned how to help themselves through participating in the program. | 0.91* | 0.87* |
| Participants have had positive change(s) after joining the program. | 0.91* | 0.87* |
| Participants have learned how to solve their problems through participating in the program. | 0.89* | 0.86* |
| Participants' behavior has become better after joining this program. | 0.86* | 0.82* |
| Those who knew the participants agree that this program has induced positive changes in them. | 0.83* | 0.78* |

*Note:* A Bonferroni adjustment to the alpha level (17 pairs of correlations) was used.

* $p < 0.003$

It is noteworthy that although the ABC approach and volunteer training and services are popular programs offered by social workers in Hong Kong, the effectiveness of these programs has not been properly documented. Although the ABC approach can help to instill novel experiences in young people who are usually not good at verbal expression and its usefulness has been endorsed by counselors[16,17,18], its long-term effect is questionable[9].

The positive evaluation findings based on the service learning programs are basically consistent with the literature that engagement in voluntary service is conducive to the positive development of adolescents[19,20]. However, as some students in Hong Kong may treat voluntary work as a vehicle for enriching their resumes, there is a need to understand their motives for joining voluntary service and the long-term effects of engaging in voluntary work in Hong Kong. In short, in view of the paucity of the research findings for these two categories of services in Hong Kong, the present findings constitute interesting additions to the literature.

The second observation is that only 15%, i.e., 32 schools, did not use the two dominant program elements in their Tier 2 Program. Those programs included promoting discipline through military march training, social skills training, promoting learning skills, promoting learning motivation, self-understanding group, and community-based oral history projects. Among the schools that adopted the nonpopular program theories, respondents in three schools (9.37%) that involved students, parents, and teachers in their Tier 2 Program gave the highest rating of 4.74 on the overall effectiveness (Table 1). It must be pointed out that the high ratings may be due to the small sample size. However, the involvement of students, parents, and teachers in specially designed programs for the school is particular suitable for students with greater psychosocial needs. In particular, the effect of involvement of parents should be further examined because parents are an essential and motivated resource in supporting positive youth development. How they can be best utilized should warrant more attention and advocacy. Moreover, 23 schools (71.8%) that involved only students had the second highest rating of 4.62 (Table 1). Further qualitative studies are necessary in order to illuminate the reasons of higher program effectiveness, and to examine the correlation between program elements and program effectiveness .

**TABLE 6**
**Comparison of the Views about the Tier 2 Program among Different Program Approaches**

| Variable | Program Approach | Mean (Standard Deviation) | F-value |
|---|---|---|---|
| Comparison between ABC approach–related programs and others | | | |
| Views about program | 1 | 4.58 (0.40) | 0.13$^{n.s.}$ |
| | 2 | 4.60 (0.41) | |
| Views about workers | 1 | 4.78 (0.39) | 0.07$^{n.s.}$ |
| | 2 | 4.80 (0.39) | |
| Perceived effectiveness of the program | 1 | 4.55 (0.42) | 0.02$^{n.s.}$ |
| | 2 | 4.54 (0.43) | |
| Global views | 1 | 4.64 (0.39) | 0.03$^{n.s.}$ |
| | 2 | 4.65 (0.39) | |
| Comparison between volunteer training and services–related programs and others | | | |
| Views about program | 3 | 4.56 (0.43) | 0.62$^{n.s.}$ |
| | 4 | 4.61 (0.36) | |
| Views about workers | 3 | 4.77 (0.41) | 0.73$^{n.s.}$ |
| | 4 | 4.81 (0.34) | |
| Perceived effectiveness of the program | 3 | 4.56 (0.45) | 0.07$^{n.s.}$ |
| | 4 | 4.54 (0.37) | |
| Global views | 3 | 4.63 (0.42) | 0.21$^{n.s.}$ |
| | 4 | 4.65 (0.34) | |
| Comparison between programs adopting two program approaches and others | | | |
| Views about program | 5 | 4.56 (0.43) | 062$^{n.s.}$ |
| | 6 | 4.61 (0.36) | |
| Views about workers | 5 | 4.77 (0.41) | 0.73$^{n.s.}$ |
| | 6 | 4.81 (0.34) | |
| Perceived effectiveness of the program | 5 | 4.55 (0.45) | 0.07$^{n.s.}$ |
| | 6 | 4.54 (0.37) | |
| Global views | 5 | 4.63 (0.42) | 0.21$^{n.s..}$ |
| | 6 | 4.65 (0.34) | |

*Note:* n.s. = nonsignificant; 1 = ABC approach–related programs (Types A and B); 2 = others (Types C and D); 3 = volunteer training and services–related programs (Types A and C); 4 = others (Types B and D); 5 = programs adopting two program approaches (Type A); 6 = programs adopting one program approach or other approaches (Types B, C, D).

Third, overall speaking, 87.87% of participants were satisfied with the services received (see Table 2). This overall rating compared favorably with the findings from two previous studies on the EIP 2005/06 for Secondary 1 students (86.82%) and EIP 2006/07 for Secondary 2 students (87.65%)[4,5]. Comparing the four types of programs, Type C received the lowest ratings in all eight indicators for program satisfaction. However, only one indicator was rated positively below 80%. It is “participants would recommend others who have similar needs to participate in the program” (79.02%). Also, the last indicator “on the whole, participants were satisfied with the service” was rated positively by 84.71% of 1,224 participants. It revealed that the Type C programs in different schools were well received.

Fourth, over 85% of participants rated positively on the eight indicators for Tier 2 Program workers, ranging from 85.47% on “the worker(s) had much interaction with participants” to 90.42% on “on the whole, participants were satisfied with the worker(s)” (see Table 3). Comparing the four types of programs, Type C received the lowest ratings in all eight indicators for program workers. However, all the ratings were positively over 83%, ranging from 83.24% on “the worker(s) had much interaction with participants” to 87.78% on “on the whole, participants were satisfied with the worker(s)”. It revealed that the social workers in different schools provided quality services. There are several plausible reasons for the comparatively low ratings: (1) the use of program involved only volunteer training and services; (2) no teachers were involved in the Type C program; (3) the average program attendance was among the lowest, i.e., 77.18% (involving students and parents) and 83.13% (involving only students) (see Table 1).

Fifth, over 80% of participants rated positively on the eight indicators for program effectiveness, i.e., ranging from 80.55 to 87.14% (see Table 4), showing that the participants were generally satisfied with the results. Since the participants were generally satisfied with the program delivery and the workers, it is not surprising to see that the perceived program effectiveness was high. However, participants in the Type A program perceived the program to be more effective than the other three types of programs. All ratings on the eight items were the highest among all categories (see Table 4).

It is noteworthy that there are several limitations of the present study. First, as only Secondary 1 (i.e., Grade 7) students were involved, further studies are needed in order to examine each program type to illuminate issues of program effectiveness, and whether or not adult participants rated differently in comparison with Secondary 1 participants. Second, only 64.43% of the program participants responded to Form C and some of the adult participants may not have completed the evaluation form. Obviously, effort should be made to understand the factors contributing to this observation. Third, as the present findings were “reconstructed” from the evaluation reports submitted by the agencies, the units of analyses were schools instead of individuals. As such, the power of the statistical analyses would become low and individual variations were lost in the process. Fourth, it is logical to ask which program seems to be most cost effective when the ultimate level of appreciation is about the same. Factors such as manpower and resources input and program output can be considered. However, due to limitations in resources, these aspects were not covered in this study. Finally, the limitations of subjective outcome evaluation utilizing quantitative findings should be realized[21,22] and the addition of qualitative findings would be helpful[23,24]. Furthermore, if resources permit, studies attempting to examine the convergence of objective outcome evaluation findings and subjective outcome evaluation findings[25] should be carried out.

## ACKNOWLEDGMENTS

The preparation for this paper and the Project P.A.T.H.S. were financially supported by The Hong Kong Jockey Club Charities Trust.

## References

1. Shek, D.T.L. (2006) Adolescent developmental issues in Hong Kong: relevance to positive youth development programs in Hong Kong. *Int. J. Adolesc. Med. Health* **18**, 341–354.
2. Shek, D.T.L. (2006) Construction of a positive youth development program in Hong Kong. *Int. J. Adolesc. Med. Health* **18(3)**, 299–302.
3. Wong, K.Y. and Lee, T.Y. (2005) Professional discourse among social workers working with at-risk adolescents in Hong Kong: risk or resilience? In *Pathways to Resilience: a Handbook of Theory, Methods, and Intervention.* Ungar, M., Ed. Sage, Thousand Oaks, CA. pp. 313–327.
4. Shek, D.T.L., Lee, T.Y., Sun, R.C.F., and Lung, D.W.M. (2008) Positive youth development programs targeting students with greater psychosocial needs: subjective outcome evaluation. *TheScientificWorldJOURNAL: TSW Holistic Health & Medicine* **8,** 73–82. DOI 10.1100/tsw.2008.3.

5. Lee, T.Y. and Tang, V.M.Y. (2008) Positive Youth Development Programs Targeting Students with Greater Psychosocial Needs: Findings based on Three Subjective Outcome Evaluation Studies. Paper presented at the 4th International Conference on Holistic Health and Medicine, University of Kentucky, Lexington, September 24–26.
6. Shek, D.T.L. and Ma, H.K. (2007) Subjective outcome evaluation of the Project P.A.T.H.S.: findings based on the program participants. *TheScientificWorldJOURNAL* **7,** 47–55. DOI 10.1100/tsw.2007.38.
7. DuBois, D. and Neville, H.A. (1996) Youth mentoring: investigation of relationship characteristics and perceived benefits. *J. Community Psychol*. **25(3),** 227–234.
8. DuBois, D.L. et al. (2002) Effectiveness of mentoring programs for youth: a meta-analytic review. *Am. J. Community Psychol*. **30(2),** 157–197.
9. Moote, G.T. and Wodarski, J.S. (1997) The acquisition of life skills through adventure-based activities and programs: a review of the literature. *Adolescence* **32(125),** 143–167.
10. Shek, D.T.L., Siu, A.M.H., Lui, J.H.Y., and Lung, D.W.M. (2006) *P.A.T.H.S. to Adulthood: a Jockey Club Youth Enhancement Scheme (Evaluation Manual).* Social Welfare Practice and Research Centre, The Chinese University of Hong Kong.
11. Schon, D.A. (1991) *The Reflective Practitioner: How Professionals Think in Action*. Avebury, Aldershot, Hants.
12. Catalano, R.F., Berglund, M.L., Ryan, J.A.M., Lonczak, H.S., and Hawkins, J.D. (2002) Positive Youth Development in the United States: Research Findings on Evaluations of Positive Youth Development Programs. Available from http://aspe.hhs.gov/hsp/PositiveYouthDev99/
13. Cheung, C.K. (2006) Experiential learning strategies for promoting adolescents' voluntarism in Hong Kong. *Child Youth Care For*. **35(1),** 57–78.
14. Fletcher, T.B. and Hinkle, J.S. (2002) Adventure based counseling: an innovation in counseling. *J. Couns. Dev*. **80,** 277–285.
15. Lee, T.Y., Shek, D.T.L., and Kwong, W.M. (2007) Chinese approaches to understanding and building resilience in at-risk populations. *Child Adolesc. Psychiatr. Clin. N. Am*. **16,** 377–392.
16. Lee, T.S. and Mak, S.W. (2002) *Adventure Counseling Handbook*. Breakthrough, Hong Kong. [Chinese]
17. Glass, J.S. and Shoffner, M.F. (2001) Adventure-based counseling in schools. *Prof. Sch. Couns*. **5,** 42–48.
18. Glass, J.S. and Myers, J.E. (2001) Combining the old and the new to help adolescents: individual psychology and adventure-based counseling. *J. Ment. Health Couns*. **23,** 104–114.
19. Leming, J.S. (2001) Integrating a structured ethical reflection curriculum into high school community service experiences: impact on students' sociomoral development. *Adolescence* **36,** 33–45.
20. Scales, P.C., Blyth, D.A., Berkas, T.H., and Kieismeier, J.C. (2000) The effects of service learning on middle school students' social responsibility and academic success. *J. Early Adolesc*. **20,** 332–358.
21. Shek, D.T.L. and Ma, H.K. (2007) Subjective outcome evaluation of the Project P.A.T.H.S.: findings based on the perspective of the program participants. *TheScientificWorldJOURNAL* **7,** 47–55. DOI 10.1100/tsw.2007.38.
22. Shek, D.T.L., Siu, A.M.H., and Lee, T.Y. (2007) Subjective outcome evaluation of the Project P.A.T.H.S.: findings based on the perspective of the program implementers. *TheScientificWorldJOURNAL* **7,** 195–203. DOI 10.1100/tsw.2007.43.
23. Shek, D.T.L. and Sun, R.C.F. (2007) Subjective outcome evaluation of the Project P.A.T.H.S.: qualitative findings based on the experiences of program implementers. *TheScientificWorldJOURNAL* **7,** 1024–1035. DOI 10.1100/tsw.2007.161.
24. Shek, D.T.L. and Sun, R.C.F. (2007) Subjective outcome evaluation of the Project P.A.T.H.S.: qualitative findings based on the experiences of program participants. *TheScientificWorldJOURNAL* **7,** 686–697. DOI 10.1100/tsw.2007.126.
25. Shek, D.T.L., Lee, T.Y., Siu, A.M.H., and Ma, H.K. (2007) Convergence of subjective outcome and objective outcome evaluation findings: insights based on the Project P.A.T.H.S. *TheScientificWorldJOURNAL* **7,** 258–267. DOI 10.1100/tsw.2007.59.
